# John Alexander Nixon

**Published:** 1951-04

**Authors:** 


### Obituary

### JOHN ALEXANDER NIXON

*C.M.G.*, M.D. Cantab., F.R.C.P. London

Colonel A. M. S.; Emeritus Professor of Medicine in the University of Bristol.

All medical colleagues in Bristol and the South West will feel a sense of loss in the passing of Professor J. A. Nixon, after a short illness, on 17th March. For very nearly fifty years he had been a notable figure in the professional world. He was the son of Robert Bell Nixon, and the grandson of Surgeon-Major Alexander Hunter, Madras Army; and was educated at Hurstpierpoint, and at Gonville and Caius College, Cambridge, where he was a Choral Exhibitioner and Tancred Student. Thence he went to St. Bartholomew's, where he was President of the Abernethian Society and Editor of the Hospital Journal. After qualifying he held house appointments at Barts' and at the Metropolitan Hospital.

In 1902 he came to the Bristol Royal Infirmary as house physician, and then senior resident officer. Having taken the

M.R.C.P., he was in 1906 elected Honorary Assistant Physician to the Royal Infirmary, with charge of dermatological cases—for in those remote days a separate " Department " had not come into being. Three years later he was elected full Physician, and shortly after F.R.C.P. During the 1914–18 war he held a commission as Temporary Colonel, A.M.S., being awarded the C.M.G. in recognition of his services in France and Germany. On resuming his consultant work he was appointed Professor of Medicine in the University of Bristol in 1924.

Some indication is given of the wide scope of his work and interests by a summary of his appointments: to the staffs of Southmead Hospital, Cossham's Memorial Hospital, and Bristol Mental Hospital; Examiner to the National University of Ireland; Inspector for the General Medical Council; President of the Bristol Medico-Chirurgical Society (1922), of the Bath and Bristol Branch of the B.M.A. (1929), and of the Association of Physicians; FitzPatrick Lecturer (R.C.M.) in 1941; and Long Fox Memorial Lecturer in 1930. But outside purely professional activities he will be remembered as a keen member of the Medical Book Society and of the Victoria Square Gardens Committee, for his active interest in the work of St. Andrew's Church, in Freemasonry, in the history of medicine and in music; and at an age when most of us have " retired " he might be found playing a sound game of tennis or cycling over the Mendips with his family.

Our readers will naturally remember him for the numerous contributions to our pages: particularly " The Influence of Food on Prevention of Disease ", and his Presidential Address on " The Debt of Medicine to the Fine Arts ". Notable among the many contributions from his pen were the *Textbook of Nutrition* (1938) and *Health and Sickness in the Merchant Navy* (1941). He was associated with the *Journal* for many years from 1912 and Editor for nearly twenty-two, during which time he produced the fifty-year index, and the *Bristol Medical School Centenary Memorial*, and sustained our fortunes through the difficult years of total war.

To his wife and family we extend our very sincere sympathies.

